# Analysis of Spinal Dysfunction in Breast Cancer Survivors with Lymphedema

**DOI:** 10.31557/APJCP.2021.22.6.1869

**Published:** 2021-06

**Authors:** Shinde Sandeep Babasaheb, Kulkarni Kajol Rajesh, Kolekar Shital Yeshwant, Sanjaykumar Patil

**Affiliations:** 1 *Department of Musculoskeletal Sciences, Faculty of Physiotherapy, Krishna Institute of Medical Sciences, Deemed to Be University, Karad, Maharashtra, India. *; 2 *Department of Obstetrics and Gynaecology, Krishna Institute of Medical Sciences, Deemed to Be University, Karad, Maharashtra, India. *

**Keywords:** Lymphedema, breast cancer survivors, spinal dysfunction, static stability, dynamic stability

## Abstract

**Background:**

To study and analyse the spinal dysfunction in breast cancer survivors with lymphedema.

**Methods:**

This study was carried out by analysing total 116 breast cancer survivor women, who were having lymphedema. Out of 116 subjects, 39 undergone radical mastectomy (RM), 39 undergone modified radical mastectomy (MRM) and 38 undergone breast conserving surgery (BCS). Thesesubjects were assessed for spinal function bytaking range of motionusing goniometer, lymphedema measurement usinginch tape, spinal stability test and functional rating index.

**Results:**

The spinal range of motion wassignificantly reduced in patients suffering from lymphedema in breast cancer survivors. The strength and endurance were significantly reduced in abdominals, extensors and lateral muscles of spine. There wasmarked effect seen on quality of life of patients assessed by using functional rating index due to spinal dysfunction in lymphedema patients.

**Conclusion:**

This study showed that there is statistically significant spinal dysfunction caused due to lymphedema in breast cancer survivors.

## Introduction

Breast cancer is the most common malignant tumour in female patients (Mangone et al., 2019). It is common in Asian population. Breast cancer is common and an estimated 1 in 7 women will develop breast cancer at some time in her life (Loh and Quek, 2011). Various form of surgeries is done for management of breast cancer patients such as radical mastectomy, modified radical mastectomy and breast conserving surgery (De GRoef et al., 2016). 

Breast cancer has one of the best surgical survival rates among the various types of cancer. It is due to its advanced screening process (Hoe et al., 1992). The mortality rate has been reduced but morbidity rates has increased. It is seen as a chronic illness with many persistent medical and non-medical problems, one of which is lymphedema (Didem et al., 2005; Ochalek, 2011).

Lymphedema is the most common chronic impairment following breast cancer surgery. Lymphedema accounts for 32% among the women diagnosed with breast cancer. It is the accumulation of protein rich fluid due to damage and obstruction in lymphatic vessels (Meraviglia, 2006). It is most commonly seen in ALND (axillary lymph node dissection) compared to SLNB (sentinel lymph node biopsy). Lymphedema can cause tightness, fullness, heaviness, paraesthesia in the affected side and disturb the posture (Mansel et al., 2006; Verbelen et al., 2014).

The spine is an important complex structure that needs to fulfil the demands of mobility and stability for the trunk and the extremities.Structurally, the spine is important to provide protection to the spinal cord. Functionally, the spine is important to maintain the postural stability i.e. dynamic stability and static stability (George and Shinde, 2019). Normal spinal alignment is important to maintain overall body posture. Dysfunction in the spine will disturb the overall posture by affecting the trunk and extremities. 

It is necessary to analyse whether the lymphedema produces any changes in the spinal stability (static and dynamic) of the patients. These efforts are directed to prevent spinal dysfunction which may be caused due to lymphedema in breast cancer survivors. So, along with the treatment of lymphedema in patients who undergone radical mastectomy, modified radical mastectomy and breast conserving surgery; the therapist and physician should assess the spinal function and provide prophylactic treatment for spine (Alande et al., 2017; Angin et al., 2014).

There are studies showing the effect of breast cancer surgeries on spine and posture. There are some studies showing effect of lymphedema in breast cancer survivors on posture mainly shoulder alignment. There are gaps in the literature regarding effect of lymphedema in breast cancer survivors on spinal function. This study will help to add more evidences on spinal postural impairment that may happendue to varying severity of lymphedema.

The information from this study is designed to meet the gaps in current literature and will provide a basis for the development of new methods for assessment, examination, intervention and prophylacticmeasures for spinal dysfunction which will lead to improvedquality of life of the patient and make the activities of daily living easy. To preventspinal dysfunction, early assessment and rehabilitation are essential. Physiotherapy will help to reduce the lymphedema, correct posture, improve mobility, strength, power, endurance and avoidance of secondary complications due to lymphedema such as chronic inflammation, cellulitis, pain, fatigue, inability to work, cosmetic deformities and a significant decrease in mobility and functional status. 

## Materials and Methods

This study was performed in a breast cancer survivor support group of Krishna institute of medical sciences deemed to be university in Karad from January 2020 toFebruary 2020.Inclusion criteria: The patients who had undergone either radical mastectomy (RM), modified radical mastectomy (MRM) and breast conserving surgery (BCS) and suffering from lymphedema. Age group was ranged from 30 to 60 years old women.

Exclusion criteria: Male breast cancer survivors and females with known case of traumatic musculoskeletal injuries, neurological deficits and patients operated for breast cancer but not suffering from lymphedema.Before initiating the study, all the participants were informed about the study procedure. A written informed consent was obtained from each participant. The study protocol was approved by the Ethical Committee [Protocol number-0132/2019-2020].

Total 116 breast cancer survivor women suffering from lymphedema, who had undergone either radical mastectomy (RM), modified radical mastectomy (MRM) and breast conserving surgery (BCS) were included in this study.

The demographic data and past surgical history were obtained from all subjects prior to the spinal function assessment.

*Data Collection Tools*

Range of motion of cervical, thoracic and lumbar region were measured by using goniometer. Flexion, extension, side flexion and rotation were assessed in cervical region and lumbar region. Flexion, extension, side flexion, were assessed of thoracic region. The purpose for assessment of range of motion was to assess the spinal function.

Lymphedema was measured of affected upper extremity, which was the side usually operated in breast cancer surgery. It was compared with the non-affected upper extremity. The lymphedema was measured using inch tape.The purpose for measuring the lymphedema was to segregate the subjects into mild, moderate, severe lymphedema. Spinal stability was assessed by using static and dynamic test for abdominals, extensors and lateral musculature. Static stability was assessed by performing the action of certain muscle group and holding the action for 20- 30 seconds was considered normal, 15-20 seconds was considered to be good, 10-15 seconds was considered to be fair, 1-10 seconds was considered to be poor and slight contraction was considered trace. Dynamic stability was assessed by performing the action of certain muscle group and repeating the action for 20-25 times was considered normal, 15-20 repetitions was considered to be good, 10-15 repetitions was considered to be fair, 1-10 repetitions was considered to be poor and slight contraction was considered trace.Activities of daily living and quality of life was assessed by using functional rating index.

*Statistical analysis*

The collected data in this study was statistically analysed using descriptive statistics as mean, average and percentage. The spinal mobility was analysed on the basis of average, which was calculated by range of motion. For lymphedema, girth is measured using inch tape and analysed on the basis of percentage of subjects suffering from spinal dysfunctions. The spinal stability and functional rating index were analysed. 

**Table 1:** Range of Motion

Cervical Range of Motion		
	Individuals with Normal range of motion	Individuals with Decreased range of motion
Flexion	37 (31.89%)	79 (68.10%)
Extension	53 (45.68%)	63 (54.31%)
Side flexion		
Right	44 (37.93%)	72 (62.06%)
Left	40 (34.48%)	76 (65.51%)
Rotation		
Right	78 (67.24%)	38 (32.75%)
Left	74 (63.79%)	42 (36.20%)
Thoracic Range of Motion		
Flexion	35 (30.17%)	81 (69.82%)
Extension	56 (48.27%)	60 (51.72%)
Side flexion		
Right	72 (62.06%)	44 (37.93%)
Left	75 (64.65%)	41 (35.34%)
Lumbar Range of Motion		
Flexion	114 (98.27%)	2 (1.72%)
Extension	83 (71.55%)	33 (28.44%)
Side flexion		
Right	103 (88.79%)	13 (11.20%)
Left	101 (87.06%)	15 (12.93%)
Rotation		
Right	114 (98.27%)	2 (1.72%)
Left	111 (95.68%)	5 (4.31%)

**Table 2:** Lymphedema Measurement

Category	Percentage of subjects	Percentage of subjects suffering from spinal dysfunction
Mild	34.48% (40)	34.88% (15)
Moderate	42.24% (49)	75.51% (37)
Severe	23.27% (27)	100% (27)

Mild lymphedema, ˂20% increase in extremity volume; Moderate lymphedema, 20-40% increase in extremity volume; Severe lymphedema, ˃40% increase in extremity volume.

**Table 3:** Spinal Stability Test (Strength Test)

Static Spinal stability test (Strength test)		
Isometric extensorStrength test		
25-30 seconds hold	Able to perform	Unable to perform
	8 subjects (6.89%)	108 subjects (93.10%)
Isometric abdominal Strength test		
25-30 seconds hold	Able to perform	Unable to perform
	22 subjects (18.96%)	94 subjects (81.03%)
Isometric Side Strength bridge test		
25-30 seconds hold	Able to perform	Unable to perform
	7 subjects (6.03%)	109 subjects (93.96%)
Dynamic spinal stability test (Endurance test)		
Dynamic extensor endurance test		
25 repetitions of dynamic extensor endurance test	Able to perform	Unable to perform
	9 subjects (7.75%)	107 subjects (92.24%)
Dynamic abdominal endurance test		
25 repetitions of dynamic abdominal endurance test	Able to perform	Unable to perform
	18 subjects (15.51%)	98 subjects (84.48%)
Dynamic horizontal Side bridge test		
25 repetitions of dynamic horizontal Side bridge test	Able to perform	Unable to perform
	23 subjects (19.82%)	93 subjects (80.17%)

**Table 4:** Functional Rating Index Scale

SR NO.	Scale	Grades				
		0	1	2	3	4
1	Pain	2 (1.72%)	13 (11.20%)	37 (31.89%)	38 (32.75%)	26 (22.41%)
2	Sleeping	2 (1.72%)	39 (33.62%)	23 (19.82%)	35 (30.17%)	17 (14.65%)
3	Personal care	0	48 (41.37%)	38 (32.75%)	27 (23.27%)	3 (2.58%)
4	Travel	7 (6.03%)	52 (44.82%)	23 (19.82%)	20 (17.24%)	3 (2.58%)
5	Work	6 (5.17%)	43 (37.06%)	24 (20.68%)	25 (21.55%)	14 (12.06%)
6	Recreation	15 (12.93%)	49 (42.24%)	33 (28.44%)	19 (16.37%)	18 (15.51%)
7	Frequency of pain	0	55 (47.41%)	31 (26.72%)	28 (24.13%)	2 (1.72%)
8	Lifting	0	39 (33.62%)	43 (37.06%)	32 (27.58%)	2 (1.72%)
9	Walking	22 (18.96%)	53 (45.68%)	20 (17.24%)	13 (11.20%)	2 (1.72%)
10	Standing	3 (2.58%)	34 (29.31%)	53 (45.68%)	24 (20.68%)	8 (6.89%)

## Results

Table no. 1) shows that range of motion of cervical, thoracic and lumbar region is decreased in the subjects. Table no. 3) shows that static and dynamic stability of spinal musculature is decreased. Table no. 4) shows that majority of subjects are having pain. Sleeping, working is severely affected. Recreation, lifting, standing is moderately affected. Personal care, travelling, walking is mildly affected.

## Discussion

In this study, the emphasizes was given to the effect of lymphedema in breast cancer survivors on spinal function. Breast cancer survivors due to various factors such as chemotherapy, breast conserving surgery (lymphectomy, lumpectomy) and radical mastectomy caused increase in postural stress which in turn produces various postural dysfunctions (Angin et al., 2014).

The conducted studyinterpreted that lymphedema causes various adverse effect on the spine. There are changes seen in the functions of thoracicregion which may lead to postural deformities such as kyphosis and scoliosis. Changes in the cervical region give rise to decreased range of motion, decreased muscle performance and impaired neural tissue mobility. Changes in the thoracolumbar region causes decrease in the range of motion, stability, endurance and overall functional capacity which is due to increase in the weight of the arm and limited use of the affected arm due to fear (Schmitz et al., 2009). Cervical and thoracic regions are more affected than the lumbar region. Lumbar region may have mild to no changes seen.

Spinal dysfunctionwas not commonly seen in mild and moderate lymphedema but was prominently observed in severe lymphedema. The motion of upper extremity was hinderedcausing pain and discomfort (Harrington et al., 2011; Bulley et al., 2012; Gillespie et al., 2018).

The static as well as dynamic stability was impaired in the subjects. Stable spine is very important to maintain the overall posture. Instability may lead to postural defects and the patient is more prone towards mechanical injuries.The overallspinalstrength and endurancewas reduced causing difficulty in activity of daily living. Activity of daily living like sleeping, personal care, travelling, walking, standing, lifting, etc was affected. The quality of life was hampered due to the frequency and intensity of pain. This may have psychological impact like depression due to the long-term treatment of breast cancer and after the treatment of cancer the debilitating effect caused by lymphedema.

Recent articles indicate that breast cancer survivors have deleterious effects on posture and musculoskeletal system such as spine alignment and increased thoracic kyphosis and upper limb dysfunction, but the effect of lymphedema on spine is not taken into consideration. There are literatures showing the change in shoulder biomechanics and alignment after breast cancer surgery which indirectly affects the spine. The whole body is interconnected by the myokinetic chain, sothe impairment in the shoulder will ultimately affect the surrounding structures. 

Postural dysfunctions can lead to chronic postural syndromes, early degenerative changes in spine causes increased economic burden on sufferers and work- related losses. So, more attention is required to be taken regarding the posture of breast cancer survivors.

Role of physiotherapy is very important to treat lymphedema and to correct the posture, improving the range of motion, decreasing the pain, improving and maintaining physical performance and promoting fitness, health, wellness. Untreated lymphedema may cause chronic inflammation, cellulitis, pain, fatigue, inability to work, cosmetic deformities and a significant decrease in mobility and functional status and use of the affected extremity.Postural stability is one of the most important components for normal function and looked after by the physiotherapist (Basar et al., 2011; Nesvold et al., 2008). Physiotherapy can prevent further deterioration in the spinal and overall posture of the body (Harrington et al., 2013). The muscle supporting the spine (abdominals and extensors) can be trained along with the treatment of lymphedema.Also strengthening the weak muscles and stretching of tight muscles after surgery. Physiotherapy will help to correct posture, improve mobility, strength, endurance, decrease pain,avoidance of secondary complications due to lymphedema and reduce the lymphedema (Bani et al., 2007; Kim et al., 2017; Jare et al., 2019; Jaju and Shinde, 2019).

The Multicomponent Exercise Program is required to counter the multiple impairments of spine (Sawant and Shinde, 2019; Shinde, 2020). The overall posture should be assessed for better treatment. The stability,strength, endurance and power of the spinal musculature should be assessed for the better prognosis.

In conclusion, On the basis of results, it is concluded that there was significant of spinal dysfunction in breast cancer survivors suffering from lymphedema. There is significant change in the spinal range of motion, decreased spinal stability and disabilities in activities of daily living.

*ABBREVIATIONS*

ALND: Axillary Lymph Node Dissection

SLNB: Sentinel Lymph Node Biopsy

RM: Radical Mastectomy

MRM: Modified Radical Mastectomy

BCS: Breast Conserving Surgery

## Author Contribution Statement

Kulkarni Kajol conducted literature review for this manuscript, developed introduction section of manuscript. Kolekar Shital conducted the discussion of the study, findings, collected data and analysed the data. Dr. Shinde Sandeep and Dr Sanjaykumar Patil provided a description of the background information, collected data and analysed the data and participated in in prescription of the manuscript, all the authors read and approved the final manuscript.

## References

[B1] Alande AA, Sagar JH, Shinde SB (2017). Effectof early physiotherapy in post-operative radical mastectomy for lymphedema. Indian J Physiother Occup Ther.

[B2] Angin S, Karadibak D, Yavuzsen T (2014). Demirbuken I. unilateral upper extremity lymphedema deteriorates the postural stability in breast cancer survivors. Contemporary Oncol.

[B3] Bani HA, Fasching PA, Lux MM (2007). Lymphedema in breast cancer survivors: assessment and information provision in a specialized breast unit. Patient Educ Couns.

[B4] Basar S, Bakar Y, Keser I (2012). Does lymphedema affect the postural stability in women after breastcancer?. Topics Geriatric Rehabilitation.

[B5] Bulley C, Coutts F, Blyth C (2012). 25 Upper limb morbidity after treatment for breast cancer: A cross-sectional study of lymphedema and function. Eur J Surg Oncol.

[B6] De GRoef A, Van Kampen M, Tieto E (2016). Arm lymphedema and upper limb impairments in sentinel node-negative breast cancer patients: A one-year follow-up study. Breast J.

[B7] Didem K, Ufuk YS, Serdar S, Zumre A (2005). The comparison of two different physiotherapy methods in treatment of lymphedema after breast surgery. Breast cancer Res Treat.

[B8] George BA, Shinde SB (2019). Effect of activity specific spinal stabilization exercises on pain and spinal mobility in lumbar spondylosis. Int J Health Sci Res.

[B9] Gillespie TC, Sayegh HE, Bruncelle CL, Danielle KM, Taghian AG (2018). Breast cancer related lymphedema: risk factors, precautionary measures, and treatments. Gland Surg.

[B10] Goti TS, Shinde SB (2020). Effect of scapular position-motion maintenance exercise programme during post traumatic shoulder immobilization. Indian J Public Health Res Develop.

[B11] Harrington S, Padau D, Battaglini C (2011). Comparison of shoulder flexibility, strength and function between breast cancer survivors and healthy participants. J Cancer Surv.

[B12] Harrington S, Padau D, Battaglini C, Michener LA (2013). Upper extremity strength and range of motion and their relationship to function in breast cancer survivors. Physiother Theory Pract.

[B13] Hoe AL, Iven D, Royle GT, Taylor I (1992). Incidence of arm swelling following axillary clearance for breast-cancer. Br J Surg.

[B14] Jaju CS, Shinde S (2019). Prevalence of peripheral neuropathy in chronic musculoskeletal oedematous conditions. Int J Physiother.

[B15] Jare NS, Shinde S, Patil S (2019). Prevalence of myofascial dysfunctions in breast cancer sur-vivors. Int J Physiother.

[B16] Kim MS, Cha YJ, Choi JD (2017). Correlation between forward head posture, respiratory functions, and respiratory accessory muscles in young adults. J Back Musculoskeletal Rehabilitat.

[B17] Kulkarni RP, Shinde SB (2020). Effect of multicomponent exercise program on selected gait and balance parameters in young obese females. J Evolut Med Dent Sci.

[B18] Loh SY, Quek KF (2011). Cancer-behaviour-coping in women with breast cancer: Effect of a cancer self-management program. Int J Appl Basic Med Res.

[B19] Mangone M, Bernetti A, Agostini F (2019). Changes in spine alignment and postural balance after breast cancer surgery: a rehabilitative point of view. Bio Res Open Access.

[B20] Mansel RE, Fallowfield L, Kissin M (2006). Randomized multicentre trial of sentinel node biopsy versus standard axillary treatment in operable breast cancer: the ALMANAC Trial. J Natl Cancer Inst.

[B21] Meraviglia M (2006). Effects of spirituality in breast cancer survivors. In Oncol Nurs Forum.

[B22] Nesvold IL, Dahl AA, Lokkevik E, Marit Mengshoel A, Fossa SD (2008). Arm and shoulder morbidity in breast cancer patients after breast conserving therapy versus mastectomy. Acta Oncol.

[B23] Ochalek K (2011). Prevention of lymphoedema. Contemp Oncol (Pozn).

[B24] Sandeep Babasaheb S, Sanjay kumar P (2020). Challenges and opportunities for breast cancer survivorship care in India during Covid-19 pandemic. Int J Res Pharm Sci.

[B25] Sawant RS, Shinde SB (2019). Effect of hydrotherapybased exercises for chronic nonspecific low back pain. Indian J Physio Ther Occup Ther.

[B26] Schmitz Kh, Ahmed Rl, Troxel A (2009). Weight lifting in women with breast-cancer–related lymphedema. N Engl J Med.

[B27] Shinde SB, Varadharajulu G (2017). Effect of therapeutic exercise programme in adults with early rheumatoid arthritis. Indian J Physiother Occupation Ther.

[B28] Shinde SB (2020). Case report on secondary complications of neck dissection surgery. J Evolut Med Dent Sci.

[B29] Stubblefield MD, Keole N (2014). Upper body pain and functional disorders in patients with breast cancer. PM R.

[B30] Verbelen H, Gebruers N, Eeckhout FM, Verlinden K, Tjalma W (2014). Shoulder and arm morbidity in sentinel node-negative breast cancer patients: a systematic review. Breast Cancer Res Treat.

